# Coreen McGuire 2020: *Measuring difference, numbering normal. Setting the standards for disability in the interwar period* und Jaipreet Virdi 2020: *Hearing Happiness. Deafness Cures in History*.

**DOI:** 10.1007/s00048-021-00321-8

**Published:** 2022-01-31

**Authors:** Robert Stock

**Affiliations:** Humboldt-Universität zu Berlin, Berlin, Deutschland

**Coreen McGuire 2020: *Measuring difference, numbering normal. Setting the standards for disability in the interwar period (= Disability History).*** Manchester: Manchester University Press, geb., 248 S., 12 Abb., £ 25,00, ISBN: 978-1-5261-4317‑4.

**Jaipreet Virdi 2020: *Hearing Happiness. Deafness Cures in History.*** Chicago: University of Chicago Press, geb., 328 S., 40 Abb., 27,50 US$, ISBN: 978-0-2266-9061‑2.

Die menschlichen Sinne sind seit der Debatte um die Dominanz des Visuellen verstärkt in den Fokus unterschiedlicher Disziplinen gerückt. Die Kritik am Okularzentrismus bedingte gewissermaßen einen *Sensory Turn*, im Zuge dessen neben dem Hören auch Riechen, Schmecken und Tasten eingehend in ihrer historischen Dimension untersucht wurden. Bei dieser Erschließung der Sinne standen ihre kulturgeschichtlichen Hintergründe wie auch sozialen Bedeutungen im Mittelpunkt. Seit einiger Zeit werden diese Ansätze durch Forschungsarbeiten komplementiert, die den engen Zusammenhang von Wissens- und Technikgeschichte mit der Produktion sinnlicher Wahrnehmungen vor allem seit dem 19. Jahrhundert und angesichts der Technowissenschaft herausarbeiten. Hierbei wird zum einen die dringliche Frage nach der wechselseitigen Bedingung der Sinne und technischer Apparaturen aufgeworfen und damit die „Natur“ der menschlichen Wahrnehmung jenseits kultureller und sozialer Rahmungen auch als technisch situiert. Zum anderen eröffnet die experimentell-technische Vermessung der Sinne den Horizont eines medizinischen und auf statistischen Werten basierenden Wissens zu sensorischem Vermögen, durch das Kategorien wie „blind“ oder „taub“ und damit auch medizinische Differenzierungen von „normal“ und „behindert“ stabilisiert werden. Dies zeigt etwa der Band *Testing Hearing. The Making of Modern Aurality* (2020) von Viktoria Tkaczyk, Mara Mills und Alexandra Hui zur Geschichte audiometrischer Tests und der modernen „Auralität“. Eine ähnlich produktive Verschränkung von Wissenschaftsgeschichte, Technikanthropologie und Disability Studies legt Coreen McGuire mit *Measuring difference, numbering normal* vor.

McGuires Buch befasst sich mit dem Zusammenhang zwischen dem Messen körperlicher Fähigkeiten und der Auffassung von Behinderung. Im Fokus stehen Medizintechnologien zum Messen der Atmung und des Hörens in der Zwischenkriegszeit im Vereinigten Königreich. Die Historikerin interessiert sich vor allem für die Dissonanz von subjektiven Erfahrungen und sogenannten objektiven Messverfahren. Letztere werden als integrale Elemente eines Prozesses verstanden, in dem wissenschaftliches Wissen über körperliche Normalwerte geprägt wurde. Problematisch seien dabei *data gaps*: Solche Lücken entstünden, da die Entwicklung „neutraler“ medizinischer Technologien häufig auf einen „neutral *male*“ (3) zurückzuführen sei.

Ein Beispiel für Datenlücken ist etwa die Vermessung des „normalen“ Hörens im Feld der Audiometrie und Telefonindustrie in der Zeit nach dem Ersten Weltkrieg, als viele Soldaten mit Hörschädigungen heimkehrten. McGuire zeigt, dass die damals erhobenen Datensets zum Normal-Hören auf Untersuchungen von gesunden, männlichen Personen basierten. Daraus resultierte ein künstlich überhöhter Wert „normaler“ Hörfähigkeit, der als akustischer Standard ins Telefonnetz implementiert wurde. In Bezug auf das vom Post Office und auf Anregung von hörbehinderten Nutzer:innen hin entwickelte „amplified telephone“ (67) argumentiert McGuire, dass sich mit dem Audiometer mechanistische Vorstellungen vom Hören durchsetzten und sich eine Medikalisierung des „Hörverlusts“ vollzog.

Andere Beispiele für „disability data gaps“ (163) sieht McGuire im Feld der künstlichen Beatmung. Ausgehend vom Spirometer skizziert sie zuerst die Schwierigkeiten bei der Messung von Atemstörungen bei Korsettträgerinnen (149) und Minenarbeitern. Die Festlegung von Standardwerten der Atemfunktion wurde bei Letzteren unter anderem im Zusammenhang mit Kompensationsforderungen gegenüber Minengesellschaften diskutiert. So stufte ein Bericht des Medical Research Council (MRC) von 1936 Daten von „gesunden“ (aber bereits geschädigten) Bergarbeitern in Süd-Wales als „normal“ ein (160). Dass subjektive Erfahrungen von Patient:innen vernachlässigt wurden, zeigt sich auch bei der Konkurrenz mechanischer Beatmungsgeräte: Der Bragg-Paul Pulsator (1933) wurde von Ingenieuren zusammen mit einem Nutzer mit progressiver Muskelatrophie für den Heimgebrauch entwickelt (180) und unter anderem bei Polio-Patient:innen eingesetzt. Auf Empfehlung des MRC und aufgrund einer Spende des Industriellen Lord Nuffield setzte sich jedoch um 1939 der Drinker Apparat – die sogenannte Eiserne Lunge – und somit eine klinische Lösung durch (184), bei der Patient:innen Atmen und Sprechen an den Rhythmus der Maschine anpassen mussten.

Anschließend an Dastons und Galisons Kritik an der „mechanischen Objektivität“ (5) entwickelt McGuire ihre These einer „mechanical epistemic injustice“ (37) bezüglich der Vermessung von defizitärem Hören und Atmen als „messbaren Pathologien“ (6). So problematisiert sie Diskriminierungen jener Gruppen, deren verkörperte Erfahrungen oft keinen Eingang in die Konzeption medizinischer Instrumente oder Technologien fanden. Dabei versucht sie auch, rezente Konzepte wie „tinkering“ (192) und Ko-Design sozio-technischer Artefakte (Bess Williamson) einzubeziehen, was aber nicht durchweg überzeugend gelingt.

Zwischen Technikgeschichte und Disability History verortet, fließen in *Hearing Happiness* von Jaipreet Virdi auch persönliche Erfahrungen als Hörgeräte-Nutzerin ein. Ausgehend von einer Kritik des Audismus (13) – das heißt medizinischen Strategien, dem Hörverlust mit Erziehung zur Lautsprache zu begegnen – erkundet Virdi die Versuche, Taubheit medizinisch und technisch zu „heilen“. Sie geht von einem Prozess „ambivalenter Medikalisierung“ (Laura Mauldin) aus und untersucht eine „Rhetorik der Heilung“ (33): Es geht um Ökonomie und Normalisierung der Hörschädigung und wie diese durch Werbemaßnahmen, medizinische Expert:innen und weitere Akteur:innen mediatisiert wurde: „the urgency to ‚fix‘ through technological intervention and shift the deaf person from citizen to consumer“ (31).

Die Historikerin beschreibt zuerst Versuche aus dem 19. Jahrhundert, Taubheit mit Quacksalberei, speziellen Diäten, der eustachischen Röhre (68) und obskuren Praktiken zu heilen. Danach wendet sie sich mit einem biografischen Ansatz „neuen Identitäten“ von Hörgeräte-Träger:innen zu, die nicht nur passive Rezipient:innen medizinischer Hilfsmittel waren, sondern auch zu kreativen Lösungsansätzen beitrugen. Des Weiteren rückt sie frühe Modelle elektrischer Hörgeräte wie das Akouphone (119) und andere elektrische „machines for deafness cures“ in den Fokus.

Während Virdi technische Entwicklungsprozesse – die Einführung von Miniatur-Vakuumröhren oder Transistoren – nur andeutet, liegt ihr Hauptaugenmerk auf visuellen Werbestrategien. An der Zeitschrift *Better Living* demonstriert die Autorin, wie Anzeigen für Sonotone-Hörgeräte Werte und Geschlechternormen der US-amerikanischen Nachkriegsgesellschaft reproduzierten: Werbefotografien zeigen erfolgreiche Geschäftsmänner und schöne Frauen einer weißen Mittelschicht, die einer Stigmatisierung als „taub“ durch miniaturisierte Hilfsmittel in Krawattennadeln, Broschen oder anderen Accessoires potenziell entgehen konnten. Teils unrealistische Werbeversprechen seitens der Hersteller bezüglich der Funktionalität führten zu heftigen Diskussionen über ethische Richtlinien für die Vermarktung von Hörgeräten, wie die Autorin hinsichtlich des Committee on Advertising Standards – gegründet von der American Society for the Hard of Hearing – darlegt (230).

Virdi betont, dass Taubsein keine Tragödie ist (265). Ihre schmerzhafte Erfahrung des Taubseins resultiere vielmehr aus dem Widerspruch zwischen Ansätzen, die das Gehör „reparieren“ möchten, und dem Anspruch, die Diversität sensorisch-kultureller Wahrnehmungsweisen anzuerkennen. Ihre situierte Perspektive auf die vielfältigen Versuche, Taubsein zu „heilen“, vermag vielschichtige Einblicke in eine Wissensgeschichte des Hörens und der Hörgeräte samt kultureller und affektiver Implikationen zu schaffen, was durch die zahlreichen Abbildungen gestützt wird. Problematisch scheint jedoch die detaillierte und oft anekdotische Herangehensweise, die mitunter Aufzählungscharakter hat.

Beide Bücher bieten eindrückliche Analysen des Hörens beziehungsweise auch des Atmens: Die technik- und medizingeschichtliche Herangehensweise McGuires eröffnet wichtige Perspektiven auf die technische Vermessung der Sinne beziehungsweise des Körpers und Normierungen mit Fokus auf Spirometrie respektive Audiometrie, Gender-Aspekte und körperliche Differenz. Durch seinen regionalen Fokus stellt das Buch eine gute Ergänzung zu anderen Studien (z. B. Mara Mills) dar, die sich auf den nordamerikanischen Kontext beziehen. Die kulturwissenschaftlich informierte Betrachtung Virdis ist hingegen als Gegenpol zu einer auf Verallgemeinerbarkeit abzielenden Medizin- oder Wissensgeschichte zu lesen. Insofern markiert *Hearing Happiness* eine engagierte Position in einem Forschungsfeld, das sich bislang auf die historische Produktion von Hören durch Experimentalanordnungen und Testregime konzentriert.

